# Prediction of Covid-19 infection severity using ABO blood group types and Rh factor

**DOI:** 10.12669/pjms.38.7.5128

**Published:** 2022

**Authors:** Syed Asim Ali Shah, Naveed Arshad, Fareena Asim, Muhammad Nadeem

**Affiliations:** 1 Dr. Syed Asim Ali Shah, FCPS (Medicine), Associate Professor, POF Hospital, Wah Cantt, Wah Medical College, NUMS Pakistan; 2 Dr. Naveed Arshad, M.Phil (Rehabilitation Sciences), Assistant Professor, Islamabad Medical and Dental College, Islamabad Pakistan; 3 Dr. Fareena Asim, MCPS (Clinical Pathology), Lecturer/Clinical Pathologist, POF Hospital, Wah Cantt, Wah Medical College, NUMS Pakistan; 4 Dr. Muhammad Nadeem, FCPS (Medicine), Professor of Medicine, Poonch Medical College, Rawalakot AJK, Pakistan

**Keywords:** ABO blood-group system, Covid-19, Rh-hr blood-group system, Severity of illness

## Abstract

**Background & Objective:**

Biological markers for the prediction of acquiring Covid-19 risk are deficient and there is a dire need of immediate research data. The objective of the study was to predict the link of ABO blood group types along with Rh factor distribution with the severity of Covid-19.

**Methods:**

This was an observational cross-sectional survey conducted in medicine department of Pakistan Ordnance Factory Hospital, Wah Cantt Pakistan, from August 2020 to December 2020 after approval of IRB. Participants tested positive for presence of Covid-19 infection by polymerase chain reaction (PCR) were included in the study. Covid-19 infection severity was measured through mild, moderate and severe disease categories and analyzed. ABO blood group and Rh subgroups data for all the Covid-19 infected patients were obtained from the laboratory section of the hospital and analyzed. Data was entered in SPSS v 26 and analyzed. Cox regression model was used to find out the severity of Covid-19.

**Results:**

Total 248 patients were included; 75% patients were male and 25% were females. The mean age of the patients was 52.77±15.58 years. A very significant association was found between ABO blood group types, Rh factor antigen and severity of Covid-19 (p=0.001). When stratified ABO, Rh antigen blood group with health status of all patients there was a very significant association between them (p=0.013). An insignificant association between male and female odds ratio of ABO blood group types but blood group B, Rh positive antigen was more susceptible in Covid-19 positive patients.

**Conclusion:**

There is a link between ABO blood group types along with Rh factor antigen (B+ and O+) with the severity of Covid-19 positive patients. ABO blood group types and Rh factor can be used as a potential marker/tool to predict the susceptibility of acquiring Covid-19 infection as well as for severity of the infection.

## INTRODUCTION

Incidence rate of Covid-19 infection is still increasing exponentially over the globe despite the availability of different vaccines to protect the infection.^1^ According to World Health Organization (WHO) report of June 2021 more than 179 million people are infected with Covid-19 and 3.8 million people are dead due to the present pandemic. Higher risk of acquiring the infection has been found to be linked with chronic diseases (e.g., lung or cardiovascular), hypertension and diabetes mellitus.^2^ Clinical severity of an existing Covid-19 infection can be determined by using CRP, ferritin and LDH but there is no known biological maker to predict the risk of acquiring the infection before the onset of the disease.^3^

ABO blood grouping system is a type of cell identification system which was discovered by Landsteiner. This system is based on the presence of blood group antigens (A & B) and their respective antibodies (Anti-A & Anti-B) in the blood. According to this system, there are four types of phenotypes accepted universally as A, B, O and AB. Rh factor determines the presence or absence of specific antigenic structures and it can be positive or negative.^4^

Few studies have reported the association between ABO blood group types and viral infections (hepatitis B, Norwalk virus), rheumatological diseases, and cancer.^5^ Antigenic determinants of ABO blood group types were found to be associated with the increased susceptibility of acquiring viral infections. SARS-COV-2 virus, major cause of deadly pandemic of Covid-19 infection, has been found to be linked with the presence of AB antigens where individuals with A/B/AB blood groups are at greater risk of getting the infection as compared to individual without AB antigens (blood group: O).^6^ This relationship of AB antigens with higher risk of acquiring infection has been reported in very few studies in literature. More studies are warranted to examine the link of ABO blood group types with this deadly viral infection and reveal the vulnerability of different blood group individuals to acquire the infection.^7^

Association of SARS-COV-1 virus with ABO blood groups has been assessed previously. Such association was first reported by Zhao et al., for SARS-COV-2 virus and indicated that individuals with A blood group are at greater risk of acquiring Covid-19 infection than other blood groups. They concluded that blood group O’ individuals are at decreased risk of getting the infection.^8^ Few reports are available in support of these findings. Despite available studies specific biological markers to predict the risk of acquiring Covid-19 infection are still lacking.

Previously available studies were carried out using the antibodies of AB antigens such as anti-A (blood group B & O), anti-B (blood group A & O) and found that Anti-A antibodies have weak association with the risk of getting the infection as compared to other antibodies.^9^ Contrasting results has been reported by Latz et al.,^10^ where individuals with B blood groups were found to be at higher risk instead of A. This recent report urges more investigations to solve the ambiguity. Moreover, to the best of our knowledge, there is no study reporting the association of Rh factor with susceptibility and severity of Covid-19 infection. Therefore, present study was designed to examine the association of ABO blood group types and Rh factor distribution with risk and severity of the Covid-19 infection along with its relationship with clinical characteristics in Covid-19 patients.

## METHODS

This observational cross-sectional study was approved by the Institutional Review Board of Pakistan Ordnance Factory (POF) Hospital Wah Cantt, Pakistan (IRB No. POFH/ERC/99053/07, Dated: 16/03/2020) and performed in accordance with the principles of declaration of Helsinki. This study was conducted at department of Medicine of POF hospital from August 2020 to December 2020. Written informed consent was taken from all enrolled patients. A total of 248 patients having Covid-19 infection and admitted in ward (IPD) or ICU were enrolled randomly for this study.

Covid-19 patients with positive polymerase chain reaction (PCR) test and reported blood group information were included in the study by random sampling while patients without this information were excluded. Medical record of all enrolled patients was reviewed and clinical features along with basic demographics, and laboratory findings [such as lab reports (Complete blood count (CBC), C-reactive proteins (CRP), Alanine transaminase (ALT), Renal function tests (RFTs), Creatine phosphokinase (CPK), Lactate dehydrogenase (LDH), ferritin, Arterial blood gases (ABG’s), D-dimers and Coagulation profile), and Radiological assessment by initial chest X-rays (CXR) and High-Resolution Computed Tomography (HRCT) chest] were noted. Patients tested positive for Covid-19 via both nasopharyngeal (NP) and throat swab were enrolled. ABO blood group and Rh factor data for all the Covid-19 infected patients were obtained from the laboratory section of the hospital and analyzed. Rh factor was determined by using anti-D and ABO blood grouping was performed by test tube method and DIAGAST reagent was used. Polymerase chain reaction (PCR) was used to detect the presence of *E* gene for Covid-19 infection in the sample and positive PCR sample was subjected to confirmation by targeting *RdRp*. Realtime PCR kits to detect the Covid-19 infection were obtained from Roche and Invitrogen.

A structured proforma was used to enter all findings. Covid-19 infection severity was measured through mild, moderate and severe disease categories and analyzed. Covid-19 severity as follows; Mild (mild clinical symptoms, no oxygen requirement, no pneumonia on chest X-rays or HRCT chest), Moderate (fever, cough and lung CT with pneumonia (lung infiltrates less than 40%), SpO_2_ greater than 93%, may have mild oxygen requirement via nasal cannula or simple face mask or low concentration via venturi mask) and Severe (symptoms of respiratory distress i.e., respiratory rate greater than 30/minutes with SpO_2_ less than 93% at rest and/or ratio of arterial oxygen partial pressure to fractional inspired oxygen less than 300mmHg (PaO_2_/FiO_2_), or may require oxygen through NRM (Non re-breathable mask), higher oxygen concentration via venturi or non-invasive or invasive ventilation, ARDS or shock).^11^ Socio-demographic factors like age, gender, hypertension, diabetes, and abovementioned clinical information of Covid-19 were also noted. For statistical analysis the data was entered in SPSS version 26. Means and standard deviations were calculated for quantitative variables like age. Frequencies were calculated for gender, hypertension and diabetes. Chi-square test and odds ratio (ORs) with 95% confidence intervals was used for ABO blood group types and Rh factor frequency in all patients. Cox regression model was used for the analysis of the association between the ABO blood group types, Rh factor and severity of disease. A p value ≤ 0.05 was considered significant.

## RESULTS

Total 248 Covid-19 positive patients were included for the study; 75.0% (n=186) were male patients and 25.0% (n=62) were females. Also, the mean age of the patients was 52.77±15.58 years. Comorbidities like hypertension (HTN), diabetes mellitus (DM), ischemic heart disease (IHD), and stroke etc., were studied. Out of 248 Covid-19 positive patients, 20.2% (n=50) were hypertensive, 15.3% (n=38) diabetes mellitus, 4.8% (n=12) had ischemic heart disease, 1.6% (n=4) were stroke patients, 1.6% (n=4) had multiple comorbidities DM/HTN/IHD and 56.5% (n=140) were no history of any comorbidity.

**Fig.1:**
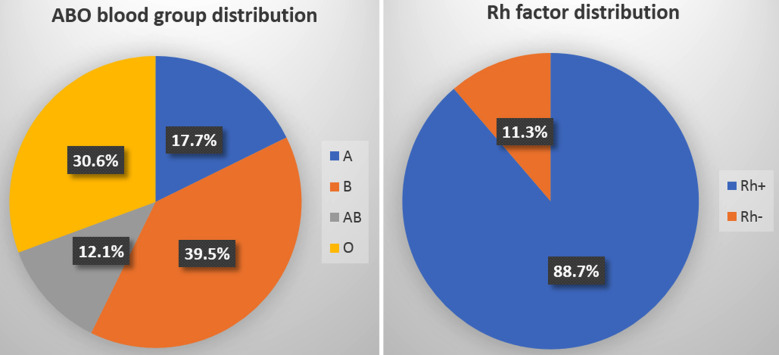
ABO blood group types and Rh factor distribution in all patients, n=248.

Percentage frequency of all blood group types of enrolled patients is shown in (Fig. 1). Briefly, frequency of A, B, AB and O blood groups was 17.7%, 39.5%, 12.1% and 30.6% respectively. Rh+ and Rh- blood group distribution was 88.7% and 11.3% respectively. Stratification analysis was performed by gender based, the association analysis revealed insignificant relation (p ≥ 0.05) between ABO blood group and Covid-19 in male and female subgroups (Table-I).

**Table I:** Stratified analysis of ABO blood group tyes by gender based, n=248.

Blood group	Gender	Frequency (%)	Chi square	p-value	OR (95% CI)
A	Male	34 (13.7%)	0.147	0.701	0.86 (0.39-1.86)
	Female	10 (4.1%)			
B	Male	70 (28.2%)	1.102	0.29	1.37 (0.76-2.44)
	Female	28 (11.2%)			
AB	Male	22 (8.9%)	0.051	0.822	1.11 (0.47-2.63)
	Female	8 (3.2%)			
O	Male	60 (24.2%)	0.911	0.340	0.73 (0.38-1.39)
	Female	16 (6.5%)			

OR: odds ratio adjusted with; CI: confidence interval.

We determine the ABO blood type with Rh blood distribution and stratification analysis was performed by severity of disease based, the association analysis revealed significant relation (p ≤ 0.05) between ABO & Rh blood group and severity of Covid-19 patients (Table-II). The severity of disease table showed, more patients of blood group B+ and blood group O+ were severe with Covid-19 as compare to other blood types.

**Table II:** Stratified analysis of ABO blood group types, Rh factor by severity of disease, n=248.

ABO blood group	Mild	Moderate	Severe	Total
A+	29 (11.7%)	10 (4%)	1 (0.4%)	40
A-	3 (1.2%)	1 (0.4%)	0	4
B+	58 (23.4%)	22 (8.9%)	8 (3.2%)	88
B-	7 (2.8%)	3 (1.2%)	0	10
AB+	18 (7.3%)	5 (2%)	1 (0.4%)	24
AB-	4 (1.6%)	2 (0.8%)	0	6
O+	42 (16.9%)	20 (8.1%)	6 (2.5%)	68
O-	5 (2%)	3 (1.2%)	0	8

Total	166 (66.9%)	66 (26.6%)	16 (6.5%)	248

Fisher’s Exact Test value = 41.547, p value = 0.001.

Also, ABO blood group types and Rh factor distribution was stratified by health status of all Covid-19 positive patients (Table-III). There were 242 (97.6%) Covid-19 positive patients alive and 6 (2.4%) patients were died. There were 4 patients of blood group B+ and 2 patients of blood group O+ were died. The association revealed significant (Fisher’s exact test = 17.854, and p=0.013), when compare ABO & Rh blood group types and Covid-19 in health status of all patients.

**Table III:** Stratified analysis of ABO blood group types, Rh factor by health status, n=248.

ABO blood group	Alive	Expired	Total
A+	40	0	40
A-	4	0	4
B+	84 (95.5%)	4 (4.5%)	88
B-	10	0	10
AB+	24	0	24
AB-	6	0	6
O+	66 (97.1%)	2 (2.9%)	68
O-	8	0	8

Total	242 (97.6%)	6 (2.4%)	248

Fisher’s Exact Test value = 17.854, p value = 0.013.

The disease severity analysis shows an average of blood group B+ (3.2%) and O+ (2.5%) had severe disease. Patients of these blood groups also have higher percentage in mild and moderate disease severity groups which is 23.4% and 16.9% in mild disease severity group respectively. In moderate disease severity group is an average of 8.9% and 8.1%, respectively. In Table-II & III, Fisher’s exact test was applied because one cell (severe & expired) has count ≤ 5.

### Table-IV Cox regression

**Table IV:** Association between ABO, Rh blood group and severity of disease, n=248.

	B	SE	Wald	df	Sig.	Exp(B)	95.0% CI for Exp(B)	

							Lower	Upper
Blood group A+	-.192	.223	.059	1	.808	1.001	.613	1.465
Blood group A-	-.519	.507	.672	1	.412	1.515	.561	4.092
Blood group B+	.192	.168	1.312	1	.252	.947	.872	1.682
Blood group B-	-1.337	.712	3.524	1	.060	1.132	.065	1.261
Blood group AB+	-.174	.251	2.211	1	.137	1.003	.888	2.377
Blood group AB-	-.902	.712	1.605	1	.205	1.320	.101	1.638
Blood group O+	.135	.182	.038	1	.846	.479	.726	1.479
Blood group O-	-1.277	.712	3.216	1	.073	1.036	.069	1.126

“Exp(B)” values showed, there is an association between blood group B+ and O+ type and severity of Covid-19 positive patients (values = 0.947 and 0.279, respectively which is ≤ 1). Other blood group types have week association.

**Fig.2:**
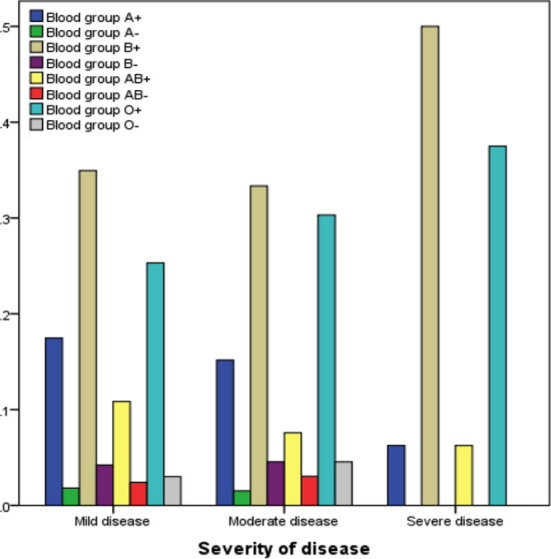
Comparison between ABO blood group types, Rh factor and severity of disease.

The comparison between ABO blood group types and Rh factor with severity of disease, Cox regression model was done and the graph shows the frequency of blood group B+ and O+ type more severe to Covid-19 positive patients is shown in Fig-2.. Other blood group types show there is a week association between them.

## DISCUSSION

Analyzing the data obtained from 248 patients of Covid-19 infection, we found that frequency of B+ and O+ blood group was higher than other blood groups. Statistical analysis revealed a significant association of ABO blood group types and Rh factor with severity of Covid-19 infection (p=0.001). It was also observed when stratified ABO, Rh blood group with health status of all patients there was a very significant association between them (p=0.013). Our results also demonstrated that there was an insignificant association between male and female odds ratio of ABO blood group types but blood group B was found to be more susceptible in Covid-19 positive patients.

Recently, several epidemiological studies had been directed to find the association between ABO blood grouping and Covid-19 infection, but there is no study performed yet on ABO blood grouping with Rh factor. Our study is unique in this regard as we performed this study on ABO blood grouping along with Rh factor such as A+, A-, B+, B-, AB+, AB-, O+ and O- with severity of disease, (mild, moderate and severe type) in our population.

Previously, a study performed by Fan et al.^12^ in Wuhan China, reported a significant association of A blood group with Covid-19 infection. They also found that females with blood group A are more susceptible (relative risk: 1.33) to acquire Covid-19 infection as compared to males. Whereas, in this study we found that males with blood group B are more susceptible to get Covid-19 infection. The odd ratio of blood group B was found to be 1.37 in this study. Another study conducted by Xiong et al.^13^ reported that Covid-19 transmission pattern in females is entirely different from males due to their different hormonal level, immune system, anatomic features and genetic makeup.

Study of this qualitative identification system of the cells is of higher importance as it is not affected by environmental factors and the presence of a particular phenotype depends on its genetic makeup only.^14^ Genes associated with blood group antigens can be a predisposing factor or a protective factor for different diseases. Susceptibility to acquire infections may increase or decrease depending on the expression of different blood group antigens. These blood group antigens play a vital role in the onset of different infections as they can act as cofactors or receptors for different types of microorganisms (e.g., viruses, bacteria, and parasites). They also play a vital role in signal transduction, cell adhesion and intracellular uptake by organization of membrane microdomains. They are also shown to completely modify the immune response of an individual to an infection.^15^,^16^

Since the onset of the pandemic, available literature on Covid-19 is increasing on daily basis. More and more researches are going on to control and prevent the pandemic situation.^17^ Zhao et al.^8^ examined the relationship of ABO blood group types with Covid-19 infection and found that individuals with A blood group are more prone to acquire infection than others. They reported that O blood group is linked with decreased risk of Covid-19 infection whereas, we concluded that blood group B (Rh+) individuals are more vulnerable to acquire Covid-19 infection and A & AB blood group represented lowest risk of the infection. We also determined the ABO blood groups associated with the severity of the infection and B+ and O+ were found to be more susceptible for the Covid-19 severity. A study conducted by Arac et al.^18^ found insignificant difference between ABO blood group types but Rh factor was strongly significant. These results support our findings that there is a strong relationship between ABO blood group types, Rh factor positive and Covid-19 infection.

Most of the studies reported that blood group A was more widespread with Covid-19 infection as compare to blood group O. Blood group O was linked with a decreased risk.^19^,^20^ But, in our study, we found that blood group B+ and O+ was more prevalent and associated with high risk.

### Limitations of the study

It is a single center and small sample size study without any control group. This study was conducted before coming the Indian strain of Covid-19, named as “Delta variant” by WHO. So, we don’t know what is the impact of ABO blood grouping and its association on “Delta variant”. In our study, we found that ABO blood grouping was statistically significant with severity of disease as well as health status of patients. Present study suggest that ABO blood group types can be a useful tool to predict the susceptibility as well as severity of Covid-19 infection. We suggest multi-center studies on larger scale community by using this tool with a control group and also include the “Delta variant” patients to see its severity with Covid-19 positive patients.

## CONCLUSION

Our study concluded that ABO blood grouping and Rh factor (mostly B+ and O+) are associated with the severity of Covid-19 infection. ABO blood group types and Rh factor may determine the greater susceptibility to the disease and also affect the course of the disease. It can be investigated in future as a potential biomarker to predict the risk of Covid-19 infection. Present study emphasizes that following the SOPs (e.g., maintain social distance, hand hygiene and use of masks) is still the most important way to prevent Covid-19 infection regardless of the presence of risk and facilitating factors.

### Authors’ Contribution:

**SAAS:** Provided concept/research design and did data collection.

**NA & MN:** did statistical analysis and manuscript writing.

**SAAS & FA:** Did editing of manuscript and project management.

**MN:** did critical revision of the manuscript for important intellectual content.

**NA & MN:** Takes the responsibility and is accountable for all aspects of the work in ensuring that questions related to the accuracy or integrity of any part of the work are appropriately investigated and resolved.

## References

[1] AL-Khikani FH (2020). Surveillance 2019 novel coronavirus (Covid-19) spreading:Is a terrifying pandemic outbreak is soon. Biomed Biotechnol Res J.

[2] Fasina FO (2020). Novel coronavirus (2019-nCoV) update:What we know and what is unknown. Asian Pacific J Tropical Med.

[3] Esref AR, Solmaz I, Akkoc H, Donmezdil S, Karahan Z, Safak KA (2020). Association between the Rh blood group and the Covid-19 susceptibility. Int J Hematol Oncol.

[4] Tobias ES, Connor M, Ferguson-Smith M (2011). Essential medical genetics. John Wiley &Sons.

[5] Arac E, Solmaz I (2019). Evaluation of blood groups in patients with anti TPO positive. Asian J Med Sci.

[6] Cheng Y, Cheng G, Chui CH, Lau FY, Chan PK, Ng MH (2005). ABO blood group and susceptibility to severe acute respiratory syndrome. JAMA.

[7] Solmaz İ, Arac S (2021). ABO blood groups in Covid-19 patients;cross-sectional study. Int J Clin Pract.

[8] Zhao J, Yang Y, Huang H, Li D, Gu D, Lu X (2020). Relationship between the ABO Blood Group and the Covid-19 Susceptibility. Clin Infect Dis.

[9] Gerard C, Maggipinto G, Minon JM (2020). Covid-19 and ABO blood group:Another viewpoint. Br J Haematol.

[10] Latz CA, DeCarlo C, Boitano L, Png CM, Patell R, Conrad MF (2020). Blood type and outcomes in patients with Covid-19. Ann Hematol.

[11] Zayed NE, Abbas A, Lutfy SM (2022). Criteria and potential predictors of severity in patients with Covid-19. Egypt J Bronchol.

[12] Fan Q, Zhang W, Li B, Li DJ, Zhang J, Zhao F (2020). Association between ABO blood group system and Covid-19 susceptibility in Wuhan. Front Cell Infect Microbiol.

[13] Xiong Q, Xu M, Zhang J, Ji M, An P, Lei H (2020). Women may play a more important role in the transmission of the corona virus disease (Covid-19) than men. Lancet.

[14] Jing W, Zhao S, Liu J, Liu M (2020). ABO blood groups and hepatitis B virus infection:a systematic review and meta-analysis. BMJ Open.

[15] Li J, Wang X, Chen J, Cai Y, Deng A, Yang M (2020). Association between ABO blood groups and risk of SARS-CoV-2 pneumonia. Br J Haematol.

[16] Cooling L (2015). Blood groups in infection and host susceptibility. Clin Microbiol Rev.

[17] Wu Y, Feng Z, Li P, Yu Q (2020). Relationship between ABO blood group distribution and clinical characteristics in patients with Covid-19. Clin Chim Acta.

[18] Arac E, Solmaz I, Samanci S (2019). ABO and Rh blood groups frequency in men, women and neonates in Diyarbakir province. Ann Med Res.

[19] Goker H, Karakulak EA, Demiroglu H, Ceylan CM, Buyukasik Y, Inkaya AC (2020). The effects of blood group types on the risk of Covid-19 infection and its clinical outcome. Turk J Med Sci.

[20] Deleers M, Breiman A, Daubie V, Maggetto C, Barreau I, Besse T (2021). Covid-19 and blood groups:ABO antibody levels may also matter. Int J Infect Dis.

